# The Role of NT-proBNP in the Diagnosis of Ventricular Arrhythmias in Patients with Systemic Sclerosis

**Published:** 2017-07

**Authors:** Lucian MURESAN, Ana PETCU, Crina MURESAN, Mirela RINZIS, Gabriel GUSETU, Dana POP, Dumitru ZDRENGHEA, Simona REDNIC

**Affiliations:** 1. Cardiology Dept. Rehabilitation Hospital, Iuliu Hatieganu University of Medicine and Pharmacy, Cluj-Napoca, Romania; 2. Rheumatology Clinic, Iuliu Hatieganu University of Medicine and Pharmacy, Cluj-Napoca, Romania; 3. Dept. of Internal Medicine, Second Medical Clinic, Iuliu Hatieganu University of Medicine and Pharmacy, Cluj-Napoca, Romania

**Keywords:** Systemic sclerosis, NT-proBNP, Ventricular arrhythmias

## Abstract

**Background:**

In patients with systemic sclerosis, NT-proBNP is a useful diagnostic marker for pulmonary hypertension and ventricular dysfunction, with important prognostic significance. The aim of this study was to assess the relationship between the NT-proBNP levels and the presence and severity of ventricular arrhythmias in patients with scleroderma.

**Methods:**

Forty consecutive patients with a diagnostic of systemic sclerosis according to the EULAR criteria admitted at the Rheumatology Clinic of Cluj-Napoca, Romania, from Jan 2014 to Apr 2014 were enrolled. Patients underwent a 12-lead ECG and a 24-hour Holter ECG monitoring for ventricular arrhythmias evaluation. Blood sample testing (including NT-proBNP level measurements), echocardiography, spirometry, chest X-ray and, when considered appropriate, high-resolution chest CT were performed.

**Results:**

Sixty percent of patients (n=24) had abnormal NT-proBNP serum levels (>125 pg/ml) and 10 patients had >100 PVC/24 h. There was a statistically significant correlation between the NT-proBNP levels and the total number of premature ventricular contractions (PVC) (r=0.445, *P*=0.006), total number of isolated PVC (r=0,493, *P*=0.002), total number of ventricular couplets (r=0.379, *P*=0.021) and the number of PVC morphologies (r=0.501, *P*=0.002). The presence of an NT-proBNP serum level >287 pg/ml had a sensitivity of 55% and a specificity of 93% in predicting the presence of complex ventricular arrhythmias on 24-hour Holter ECG monitoring.

**Conclusion:**

NT-proBNP levels could become a useful ventricular arrhythmia marker for assessing the arrhythmic risk in patients with systemic sclerosis.

## Introduction

N-terminal pro B-type natriuretic peptide (NT-proBNP) is a natriuretic peptide secreted by atria and ventricles and released into circulation because of increased wall tension (1, 2). The most common cause of increased serum levels of NT-proBNP is congestive heart failure (3–5). Other causes of increased NT-proBNP levels include left ventricular systemic dysfunction (6), acute coronary syndromes (7–9), percutaneous coronary interventions (10), cardiac surgery (11), heart transplantation (12), left ventricular hypertrophy (13), right ventricular dysfunction secondary to pulmonary diseases (pulmonary embolism, pulmonary hypertension, chronic lung disease and respiratory failure) (14, 15), valvular heart disease (especially mitral and aortic) (16, 17), certain cardiac inflammatory (18) or infectious diseases (19), endocrinology diseases (20) and high output states (21). NT-proBNP is a very good marker of cardiac function and is a strong prognostic parameter in patients with coronary heart disease and heart failure (2, 22).

In patients with scleroderma, increased levels of NT-proBNP have been observed in patients with pulmonary arterial hypertension and/or heart failure (23–25). In these patients, NT-proBNP levels are strong predictors of survival (26). The relationship between NT-proBNP levels and ventricular arrhythmias in patients with scleroderma needs more clarification. In other populations of patients, there is growing evidence linking increased levels of NT-proBNP with the occurrence of ventricular arrhythmias, both in patients with a severely reduced LV ejection fraction (27, 28) and a normal ejection fraction (29). In ICD carriers implanted in primary prevention of sudden cardiac death, increased baseline NT-proBNP levels were shown to be independently associated with the risk of developing sustained ventricular arrhythmias (27).

The aim of the present study was to assess the relationship between the NT-proBNP levels and the presence and severity of ventricular arrhythmias in patients with scleroderma.

## Materials and Methods

### Patient population and study protocol

The studied population consisted of 48 consecutive patients with systemic sclerosis, both the diffuse cutaneous form and the limited cutaneous form, diagnosed according to the EULAR criteria, admitted at the Rheumatology Clinic of Cluj-Napoca, Romania, from Jan 2014 to Apr 2014. All patients gave informed consent before entering the study.

The examination protocol included a complete physical exam, blood sample testing, a 12-lead ECG, transthoracic Doppler echocardiography and a 24-hour Holter ECG monitoring. All patients also underwent spirometry testing and a standard chest X-ray. In cases where pulmonary fibrosis was suspected based on the patient’s symptoms, abnormal chest X-ray and spirometry results, a high-resolution chest computed tomography (CT) was also performed.

Clinical data were collected from the patient’s clinical records. The 12 lead ECG, 24-hour Holter ECG results, and the transthoracic echocardiography examinations were interpreted by a full-time cardiologist. The chest X-ray and, where performed, the chest CT results were interpreted by a single experienced radiologist.

Based on the above-mentioned paraclinical examinations, patients diagnosed with conditions that could explain an increased level of NT-proBNP were excluded from the study, as follows: 2 patients with moderate pulmonary hyper-tension, 1 patient with severe pulmonary hyper-tension, 1 patient with severe pulmonary hyper-tension and severe left ventricular (LV) hypertrophy, 1 patient with moderate LV hypertrophy, 1 patient with moderate LV hypertrophy and moderate mitral regurgitation, 1 patient with moderate mitral regurgitation and severe pulmonary hyper-tension and 1 patient with mild LV hypertrophy. The remaining 40 patients represented the studied population.

### Evaluation of patients

#### Laboratory analyses

Blood sample testing included a complete blood count, erythrocyte sedimentation rate (ESR), blood urea nitrogen (BUN), creatinine, electrolytes (Na^+^, K^+^, Ca^2+,^ Cl^−^), glycemia, total cholesterol, LDL-cholesterol, HDL-cholesterol, triglycerides, uric acid, coagulation parameters (Quick time, INR, activated partial thromboplastin time), GOT, GPT, alkaline phosphatase, gamma-glutamyl transferase, bilirubin, total protein levels and albumin. Complement levels (C3, C4), circulating immune complexes (CIC), IgA, IgG, IgM, rheumatoid factor, antinuclear antibodies and anti-topoisomerase I levels were also measured. Finally, NT-proBNP serum levels (pg/ml) were measured in all patients, with a level of < 125 pg/ml being considered normal.

#### The 12 lead ECG

All 12 lead ECGs were recorded using an Esaote P8000 electrocardiograph at a speed of 25 mm/s, with an ECG amplifier sensitivity of 10 mm/mV. The assessed parameters were rhythm, heart rate, QRS axis, the presence of hypertrophy (both atrial and ventricular), myocardial ischemia, the PR interval, the QRS complex duration, the QT and the QTc interval.

Bradycardia was defined as a heart rate of < 60 bpm while tachycardia as a heart rate of > 100 bpm.

Ischemia was defined as the presence of negative T waves, ST depression of ≥1 mm or the presence of Q waves in at least 2 contiguous leads. A QTc over 440 ms for males and over 460 ms for females was considered prolonged.

#### Holter ECG monitoring

All recordings were made using a 7-lead BTL CardioPoint H600 device, with a 2000Hz sampling frequency and 16-bit digital resolution.

The assessed parameters were maximum, average and minimum heart rate, average heart rate while awake, average heart rate while asleep, the presence of supraventricular and ventricular arrhythmias, QT and corrected QT interval.

Bradycardia was defined as an average heart rate of < 60 bpm while awake and tachycardia as a heart rate of > 100 bpm while awake or asleep. All premature atrial contractions (PAC) and premature ventricular contractions (PVC) were recorded. “Significant ventricular arrhythmia” was defined as the presence of > 100 PVC / 24 h.

#### Echocardiography

All transthoracic echocardiographic examinations were carried out using an Esaote MyLab^TM^ X-View 50 machine, with a 7.5 - 10 MHz transducer. The assessed parameters were chamber size and wall thickness, systolic and diastolic function of the left ventricle (LV), systolic function of the right ventricle (RV), left-sided filling pressures, global and regional motion abnormalities, systolic, mean and diastolic pulmonary aterial pressure (sPAP, mPAP, dPAP), the presence of pericardial effusion, the presence of valve disease (stenosis and regurgitations).

LV hypertrophy was defined as increased thickness of IVS and PW (>11 mm), LV dilation as an end-diastolic dimension >60 mm, end systolic dimension >40 mm, RV dilation as a diameter of the RV >26 mm in the parasternal long axis (PLAX) view, LV systolic dysfunction as an ejection fraction of <50%, RV systolic dysfunction as TAPSE <17 mm.

Pulmonary hypertension was defined as mild if sPAP was 35–49 mmHg, moderate if between 50–69 mmHg and severe if ≥70 mmHg.

#### Statistical analysis

Statistical analysis was performed using the Statistical Package for the Social Sciences (SPSS Inc. Chicago, Illinois) ver. 20. Descriptive statistics were used to summarize patients’ characteristics. Normality was assessed for all continuous variables using the Shapiro Wilk test. When the assumption held, results were expressed as mean ± standard deviation (SD) or otherwise by median ± interquartile range. Categorical variables were presented as counts and proportions (percentage).

The Chi-square test was used to compare categorical features of the different scleroderma subgroups of patients. According to the distribution of data (normal or non-normal), the t-test for independent samples or Mann-Whitney U test were used to compare several characteristics of different scleroderma subgroups. Spearman’s correlation coefficients were used to assess the relationship between the NT-proBNP serum levels and different characteristics of ventricular arrhythmias. Receiver operating characteristic (ROC) curves were used to analyze the accuracy of Holter ECG parameters in predicting the existence of elevated serum NT-proBNP levels, and of elevated NT-proBNP levels in predicting the presence of frequent PVC and complex ventricular arrhythmia.

A *P*-value of < 0.05 was considered statistically significant.

## Results

### General clinical and paraclinical characteristics of the patients

When compared according to the scleroderma subtype, the main differences between patients with diffuse cutaneous scleroderma and limited cutaneous scleroderma were related to the skin score (17 ± 16 vs 4 ± 16, *P*=0.02) and disease duration: onset of Raynaud’s phenomenon (3 ± 6 vs. 10.5 ± 14, *P*=0.0048) and non-Raynaud’s phenomena (1.24 ± 1.4 yr vs 10.5 ± 14 yr, *P*=0.0051). Patients with the diffuse form of the disease also had a significantly lower prevalence of LV diastolic dysfunction (18.75% vs. 54.1%, *P*=0.02) and a significantly higher level of NT-proBNP serum level (232.5 ± 16 vs. 135.5 ± 16, *P*=0.04).

There were no statistically significant differences on the presence of relevant associated diseases, cardiovascular diseases, left ventricular systolic function, mild pulmonary hypertension, pericardial effusion, right ventricular dilation, presence of mild valvular disease and cardiovascular medication used. The general characteristics of the patients’ are presented in Table 1.

**Table 1:** General characteristics of the patients according to the scleroderma subtype

**Patient characteristic**	**Diffuse cutaneous scleroderma**	**Limited cutaneous scleroderma**	**Total**
**Patient number n (%)**	16 (40)	24 (60)	40 (100)
**Gender, female n (%)**	14 (87.5)	23 (95.83)	37 (92.5)
**Mean age (years)**	46.13 ± 13.13	52.67 ± 10.9	50.05 ± 12.12
**Disease characteristics**			
• Skin score, mean (range)	**17 (3–25)**	**4 (0–21)**	**9 (0–25)**
• Onset of Raynaud’s phenomenon (years) (mean ± std dev)	**3 ± 6**	**10.5 ± 14**	**9 ± 7**
• Onset of non-Raynaud’s phenomena (years) (mean ± std dev)	**1.24 ± 1.4**	**9.5 ± 4.5**	**6 ± 8.37**
• Auto-antibodies, n (%)	2 (12.5)	2 (8.33)	4 (10)
○ ANA negative			
**Associated conditions: n (%)**			
• Pulmonary fibrosis	10 (62.5)	12 (50)	22 (55)
• Abnormal spirometry results			
○ Obstructive pattern	4 (25)	11 (45.83)	15 (37.5)
○ Restrictive pattern	3 (18.75)	5 (20.83)	8 (20)
• Anemia	3 (18.75)	4 (16.66)	7 (17.5)
• Thyroid dysfunction	2 (12.5)	5 (20.83)	7 (17.5)
**Cardiovascular diseases: n (%)**			
• Arterial Hypertension	1 (6.25)	6 (25)	7 (17.5)
• Congestive Heart Failure	0 (0)	0 (0)	0 (0)
• Atrial fibrillation	0 (0)	0 (0)	0 (0)
• Dyslipidemia	8 (50)	9 (37.5)	17 (42.5)
**Pulmonary Hypertension: n (%)**	3 (18.75)	7 (29.16)	10 (25)
• Mild	3 (18.75)	7 (29.16)	10 (25)
• Moderate	0 (0)	0 (0)	0 (0)
• Severe	0 (0)	0 (0)	0 (0)
**Pericardial Effusion: n (%)**	2 (12.5)	0 (0)	2 (5)
•Mild	0 (0)	0 (0)	0 (0)
•Moderate	2 (12.5)	0 (0)	2 (5)
•Severe	0 (0)	0 (0)	0 (0)
**Left Ventricular Systolic function**			
• <50%, n (%)	0 (0)	0 (0)	0 (0)
• %, Mean ± std dev	62.63 ± 5.28	64.45 ± 8.26	63.68 ± 7.13
**Left Ventricular Diastolic dysfunction: n (%)**	**3 (18.75)**	**13 (54.16)**	**16 (40)**
• Impaired Relaxation	**3 (18.75)**	**13 (54.16)**	**16 (40)**
• Pseudonormal	0 (0)	0 (0)	0 (0)
• Restrictive filling	0 (0)	0 (0)	0 (0)
**Valve Disease (moderate or severe)**	0 (0)	0 (0)	(0)
• Mitral Regurgitation	0 (0)	0 (0)	(0)
• Aortic Regurgitation	0 (0)	0 (0)	(0)
• Tricuspid Regurgitation	0 (0)	0 (0)	(0)
**Hypertrophy / Dilation (echocardiography): n (%)**			
• Left Ventricular Hypertrophy (mild)	1 (6.25)	3 (12.5)	4 (10)
• Right Ventricular Dilation (mild)	2 (12.5)	2 (8.33)	4 (10)
• Left Atrial Dilation	1 (6.25)	1 (4.16)	2 (5)
• Right Atrial Dilation	NA	NA	NA
**NT pro BNP (pg/ml)**	**232.5 ± 16**	**135.5 ± 16**	**162 ± 168.5**
**Medication: n (%)**			
• Beta blockers	1 (6.25)	1 (4.16)	2 (5)
• ACE inhibitors/ARBs	2 (12.5)	6 (25)	8 (20)
• Calcium Channel Blockers	6 (37.5)	9 (37.5)	15 (37.5)
• PAH-specific medication	0 (0)	0 (0)	0 (0)

ANA=Antinuclear antibodies; ACE Inhibitors=Angiotensin Converting Enzyme Inhibitors; ABRs=Angiotensin Receptor Blockers; PAH=Pulmonary arterial Hypertension; NA=not available.

### Holter ECG findings

The main 24-hour Holter ECG findings are summarized in Table 2.

**Table 2:** Holter ECG findings in the studied population according to the scleroderma subtype

**Findings**	**Diffuse cutaneous scleroderma (n=16)**	**Limited cutaneous scleroderma (n=24)**	**All scleroderma patients (n=40)**
**Sinus rhythm**			
Maximum heart rate	138.75 ± 15.36	135.6 ± 23.10	137 ± 19.84
Average heart rate	**82.31 ± 9.77**	**75.24 ± 8.50**	**78.3 ± 9.62**
Minimum heart rate	59.75 ± 8.82	55.95 ± 10.99	57.6 ± 10.16
**Supraventricular arrhythmias**			
Total	62 ± 111	21 ± 57	40 ± 98
Isolated PAC	47 ± 97	21 ± 47	28 ± 75
Coupled PAC	2 ± 5	0 ± 3	1 ± 3
Triplets	0 ± 1	0 ± 1	0 ± 1
Runs of PAC	0 ± 1	0 ± 1	0 ± 1
Atrial fibrillation, n (%)	0 (0)	0 (0)	0 (0)
**Ventricular arrhythmias**			
Total	11 ± 156	4 ± 80	5 ± 103
Isolated PVC	10 ± 151	1 ± 18	2 ± 81
Coupled PVC	0 ± 1.2	0 ± 0	0 ± 0
Number of morphologies	1 ± 2.2	1 ± 2	1 ± 2

*P*<0.05 for all variables. PAC=Premature Atrial Contraction; PVC=Premature Ventricular Contraction; VT=Ventricular Tachycardia

All but two patients presented some type of supraventricular or ventricular arrhythmia. However, >100 PAC or >100 PVC/24 h were present in 40% of patients (n=16): >100 PAC in 25% of patients (n=10) and >100 PVC in 25% of patients (n=10). Four patients (n=10%) had both >100 PVC and > 100 PAC / 24 h.

Regarding the type of ventricular arrhythmias, most patients presented isolated PVC. There were 37.5% of patients with diffuse cutaneous scleroderma (n=6) who presented ventricular couplets, compared to 4.1% of patients with limited cutaneous scleroderma (n=1). Only one patient with diffuse scleroderma presented non-sustained VT, compared to no patient with limited scleroderma. There were no patients with sustained episodes of VT in either subgroup.

There were no statistically significant differences between patients with diffuse cutaneous scleroderma and limited cutaneous scleroderma in what concerns sinus rhythm parameters, number and complexity of supraventricular and ventricular arrhythmias, except for a higher average heart rate in patients with the diffuse form of the disease (82.31 ± 9.77 bpm vs 75.24 ± 8.5) (Table 2), despite an equally low use of beta blockers in each subgroup.

Characteristics of scleroderma patients according to serum NT-proBNP levels Sixty percent of patients (n=24) had increased NT-proBNP serum levels (>125 pg/ml).

The median NT-proBNP serum level was 51 ± 60 pg/ml in patients with normal NT-proBNP levels, compared to 162 ± 168.5 pg/ml in patients with elevated NT-proBNP levels, *P*<0.0001.

When compared to patients with normal serum NT-proBNP levels, patients with elevated NT-proBNP levels had a significantly higher prevalence of pulmonary fibrosis (12 patients vs. 2 patients, *P*=0.0014) and a significantly higher prevalence of dyslipidemia (11 patients vs. 1 patient, *P*=0.007).

A trend towards a higher number of PVC / 24 h was identified in patients with elevated NT-proBNP serum levels compared to patients with normal NT-proBNP levels, but this trend did not reach statistical significance (15 ± 103 vs. 1.5 ± 21, *P*=0.07).

There were no statistically significant differences in what concerns the type of scleroderma, the presence of other relevant associated diseases, cardiovascular diseases, left ventricular systolic and diastolic function, right ventricular systolic function, pulmonary hypertension, pericardial effusion and cardiovascular medication used (Table 3).

**Table 3:** General characteristics of scleroderma patients according to the NT-proBNP serum levels

**Patient characteristic**	**Normal serum NT-proBNP levels (<125 pg/ml)**	**Increased serum NT-proBNP levels (>125 pg/ml)**	**Total**
**Patient number n (%)**	16 (40)	24 (60)	40 (100)
**Gender, female n (%)**	15 (93.75)	22 (91.66)	37 (92.5)
**Mean age (years)**	47.44 ± 9.91	51.79 ± 13.31	50.05 ± 12.12
**Disease characteristics**			
• Disease subtype			
○ Diffuse Cutaneous, n (%)	4 (25)	12 (50)	16 (40)
○ Limited Cutaneous, n (%)	12 (75)	12 (50)	24 (60)
• Skin score, mean (range)			
• Onset of Raynaud’s phenomenon (years) (mean ± std dev)	12 (0–25)	5 (0–22)	9 (0–25)
• Onset of non-Raynaud’s phenomenon (years) (mean ± std dev)	10 ± 4.5	6 ± 7.75	9 ± 7
• Auto-antibodies, n (%)	7 ± 15	6 ± 8.5	6 ± 8.37
○ ANA negative	1 (6.25)	3 (12.5)	4 (10)
**Associated conditions: n (%)**			
• Pulmonary fibrosis	**2 (12.5)**	**12 (50)**	**22 (55)**
• Abnormal spirometry results			
○ Obstructive pattern	6 (37.5)	9 (37.5)	15 (37.5)
○ Restrictive pattern	4 (25)	3 (12.5)	8 (20)
• Anemia	1 (6.25)	4 (16.66)	7 (17.5)
• Thyroid dysfunction	2 (12.5)	5 (20.83)	7 (17.5)
**Cardiovascular diseases: n (%)**			
• Arterial Hypertension	2 (12.5)	5 (20.83)	7 (17.5)
• Congestive Heart Failure	0 (0)	0 (0)	0 (0)
• Atrial fibrillation	0 (0)	0 (0)	0 (0)
• Dyslipidemia	**1 (6.25)**	**11 (45.83)**	**17 (42.5)**
**Pulmonary Hypertension: n (%)**	6 (37.5)	5 (20.83)	10 (25)
• Mild	6 (37.5)	5 (20.83)	10 (25)
• Moderate	0 (0)	0 (0)	0 (0)
• Severe	0 (0)	0 (0)	0 (0)
**Pericardial Effusion: n (%)**	1 (6.25)	1 (4.16)	2 (5)
• Mild	0 (0)	0 (0)	0 (0)
• Moderate	1 (6.25)	1 (4.16)	2 (5)
• Severe	0 (0)	0 (0)	0 (0)
**Left Ventricular Systolic function**			
• <50%, n (%)	0 (0)	0 (0)	0 (0)
• %, Mean ± std dev	62.93 ± 6.63	64.17 ± 7.54	63.68 ± 7.13
**Left Ventricular Diastolic dysfunction: n (%)**	6 (37.5)	10 (41.6)	16 (40)
• Impaired Relaxation	6 (37.5)	10 (41.6)	16 (40)
• Pseudonormal	0 (0)	0 (0)	0 (0)
• Restrictive filling	0 (0)	0 (0)	0 (0)
**Valve Disease (moderate or severe)**	0 (0)	0 (0)	(0)
• Mitral Regurgitation	0 (0)	0 (0)	(0)
• Aortic Regurgitation	0 (0)	0 (0)	(0)
• Tricuspid Regurgitation	0 (0)	0 (0)	(0)
**Hypertrophy / Dilation (echocardiography): n (%)**			
• Left Ventricular Hypertrophy (mild)	2 (12.5)	2 (8.33)	4 (10)
• Right Ventricular Dilation (mild)	0 (0)	4 (16.66)	4 (10)
• Left Atrial Dilation	1 (6.25)	1 (4.16)	2 (5)
• Right Atrial Dilation	NA	NA	NA
**NT pro BNP (pg/ml)**	**51 ± 60.67**	**238 ± 226**	**162 ± 168.5**
**Medication: n (%)**			
• Beta blockers	0 (0)	2 (8.33)	2 (5)
• ACE inhibitors/ARBs	3 (18.75)	5 (20.83)	8 (20)
• Calcium Channel Blockers	7 (43.75)	8 (33.33)	15 (37.5)
• PAH-specific medication	0 (0)	0 (0)	0 (0)

ANA=Antinuclear antibodies; ACE Inhibitors=Angiotensin Converting Enzyme Inhibitors; ABRs=Angiotensin Receptor Blockers; PAH=Pulmonary arterial Hypertension; NA=not available.

### Relationship between NT-proBNP serum levels and ventricular arrhythmias

There was a statistically significant correlation between the NT-proBNP levels and several Holter ECG parameters: total number of PVC (r=0.445, *P*=0.006), total number of isolated PVC (r=0,493, *P*=0.002), total number of ventricular couplets (r=0.379, *P*=0.021) and the number of PVC morphologies (r=0.501, *P*=0.002).

The presence of a NT-proBNP serum level >287 pg/ml had a sensitivity of 50% and a specificity of 93% with an area under the curve (AUC) of 0.713 in predicting a number > 100 PVC/24 h on the Holter ECG monitoring, a sensitivity of 55% and a specificity of 93% (AUC=0.758) in predicting complex ventricular arrhythmias (ventricular bigeminy, couplets or triplets) and a sensitivity of 35% and a specificity of 93% (AUC=0.801) in predicting the presence of polymorphic PVC.

On the other hand, a number of > 117 PVC/24 h had a sensitivity of 30% and a specificity of 85% for predicting elevated serum NT-proBNP levels, with an area under the curve of 0.679. Patients with >117 PVC/24 h had significantly higher levels of NT-proBNP serum levels, compared to patients with <117 PVC/24 h: 291±792 pg/ml vs. 148±163 pg/ml, *P*=0.012.

## Discussion

We assessed the relationship between the serum levels of NT-proBNP and the types and severity of ventricular arrhythmias in patients with systemic sclerosis. The main findings of this study can be summarized as follows: 1) a significant correlation between the NT-proBNP levels and the burden and complexity of ventricular arrhythmias on the 24-hour Holter ECG monitoring; 2) patients with elevated NT-proBNP levels tend to have a higher number of PVC on the 24-hour Holter ECG monitoring compared to patients with normal NT-proBNP levels; and 3) elevated NT-proBNP levels (>287 pg/ml) are able to predict the presence of frequent and complex ventricular arrhythmias with a high specificity.

NT-proBNP is a cardiac peptide that has an important role in the screening and diagnosis of heart failure (30). Levels are usually increased in patients with asymptomatic or symptomatic left ventricular dysfunction. It also has an important prognostic role in predicting the outcome of patients with decompensated heart failure (31). Higher NT-proBNP levels can also be found in patients with right heart failure or pulmonary hypertension (14, 15, 32). Increased levels of NT-proBNP can also found in patients with arrhythmias. The currently existing studies were mainly conducted in patients with supraventricular arrhythmias, most of them in patients with atrial fibrillation. The relationship between NT-proBNP and atrial fibrillation was studied on a large population of 5445 individuals and found that on NT-proBNP was a remarkable predictor of incident atrial fibrillation, independent of any other risk factor (33). In their study on 215 patients undergoing elective coronary artery bypass graft (CABG), using multivariate analysis, NT-proBNP levels correlated independently with the post-operative occurrence of atrial fibrillation (34). A study was conducted on 40 patients with atrial fibrillation undergoing electrical cardioversion and found that NT-proBNP levels above 1707 pg/ml had a specificity of 92% and a sensitivity of 36% in predicting recurrence of atrial fibrillation 6 months after a successful cardioversion (35).

Concerning ventricular arrhythmias, there is evidence linking increased levels of NT-proBNP with the occurrence of ventricular arrhythmias, both in patients with a severely reduced LV ejection fraction (27, 28) and a normal ejection fraction (29). In their study conducted in patients implanted with an implantable cardioverter defibrillator (ICD) for the primary prevention of sudden cardiac death, elevated baseline NT-proBNP levels were independently associated with the risk of developing ventricular arrhythmias (27). A study was conducted on 30 patients with dilative cardiomyopathy and an ejection fraction of the left ventricle ≤ 40% and showed that elevated NT-proBNP levels significantly correlated with the occurrence of symptomatic ventricular arrhythmias (28). Fifty-two patients were studied with PVC but no manifestations of heart failure and no digoxin or beta-blocker therapy and showed that patients with PVC in LOWN class III and IV had BNP concentrations triple than those in LOWN class I and II (57.2 versus 18.1 pg/mL, *P*<0.01) and suggested that the BNP elevation could be a response to abnormal wall stress from the severe ventricular arrhythmias (29).

In patients with systemic sclerosis, the significance of increased NT-proBNP levels is presently attributed mainly to the concomitant presence of pulmonary arterial hypertension or right heart failure (23, 24). Indeed, increased levels of NT-proBNP can predict the occurrence of pulmonary hypertension in patients with systemic sclerosis (24). However, elevated levels of NT-proBNP in scleroderma patients can have other significances. A significant correlation was found between the serum levels of NT-proBNP and the modified Rodnan Skin score, systolic pulmonary artery pressure and histopathological skin thickness score (36).

Despite all the evidence suggesting a relationship between higher NT-proBNP / BNP and arrhythmias in other populations of patients, data in patients with systemic sclerosis is scarce.

In one study conducted on 49 patients with systemic sclerosis, BNP was the only independent predictor of incident AF in patients with systemic sclerosis (37). During a mean follow-up of 72 ± 24 months, the incidence of atrial fibrillation was high (36.7%), especially in the presence of LV diastolic dysfunction with LA mechanical overload and elevated BNP levels.

There are no previous studies evaluating the significance of elevated NT-proBNP values in patients with systemic sclerosis and ventricular arrhythmias. We found that patients with a higher number of PVC on the 24-hour Holter ECG monitoring had increased serum levels of natriuretic peptides (29). However, in our study, we measured NT-proBNP levels, whereas BNP levels were measured in their study. Nevertheless, there are other similarities between the 2 studies: both studies included patients with no manifestations of heart failure; the ejection fraction in their population of patients was 65.2% in one subgroup (LOWN class I and II) and 62.1% in the other (LOWN class III and IV), comparable to 63.68% in our population of patients.

In the present study, patients with elevated NT-proBNP levels had a higher number of PVC on the 24-hour Holter ECG monitoring compared to patients with normal NT-proBNP levels. This finding might have an important prognostic role (27), elevated NT-proBNP levels - in the upper 50th percentile - were the strongest predictor of appropriate ICD therapy in their population of patients. Our finding that elevated NT-proBNP levels >287 pg/ml are able to predict with a high specificity the presence of frequent and complex ventricular arrhythmias might be useful for future studies stratifying the arrhythmic risk in patients with systemic sclerosis and in helping to identify suitable candidates for ICD implantation.

The most significant limitation of the present study is the small number of patients included. In the absence of a large enough number of subjects included in the study, the results of this paper should be interpreted with caution. Another limitation is the relatively weak correlations between NT-proBNP levels and the number of PVC on Holter ECG monitoring. This might also be related to the small number of patients included.

The inclusion of patients with more severe ventricular arrhythmias (non-sustained ventricular tachycardia) and with a higher arrhythmia burden might have yielded different correlations with the serum NT-proBNP levels.

## Conclusion

Ventricular arrhythmias are common in patients with systemic sclerosis. Patients with elevated NT-proBNP levels tend to have a more important ventricular arrhythmia burden, regardless of comparable left ventricular ejection fraction values, systolic pulmonary artery pressure and right ventricle size on echocardiography. In the absence of at least moderate pulmonary hypertension or ventricular dysfunction, an NT-proBNP level > 287 pg/ml is highly specific for the presence of frequent and complex ventricular arrhythmias. Therefore, NT-proBNP could become a useful ventricular arrhythmia marker for identifying patients who need Holter ECG monitoring and possible referral to a cardiac electrophysiologist.

## Ethical considerations

Ethical issues (Including plagiarism, informed consent, misconduct, data fabrication and/or falsification, double publication and/or submission, redundancy, etc.) have been completely observed by the authors.
